# Modulation of oxidative stress/NMDA/nitric oxide pathway by topiramate attenuates morphine dependence in mice

**DOI:** 10.1016/j.heliyon.2024.e40584

**Published:** 2024-11-22

**Authors:** Shabir Hussain, Haji Bahadar, Muhammad Imran Khan, Neelum Gul Qazi, Shabnum Gul Wazir, Habab Ali Ahmad

**Affiliations:** a Department of Pharmacology, Institute of Basic Medical Sciences, Khyber Medical University, Peshawar, Khyber Pakhtunkhwa, Pakistan; b Institute of Pharmaceutical Sciences, Khyber Medical University, Khyber Pakhtunkhwa, Pakistan; c Department of Biomedical Sciences, Pak Austria Fachhochschule: Institute of Applied Sciences and Technology, Haripur, Khyber Pakhtunkhwa, Pakistan; d Department of Pharmacy, Iqra University, Islamabad, Pakistan; e Frontier Medical and Dental College, Abbottabad, Khyber Pakhtunkhwa, Pakistan

**Keywords:** Morphine, NMDA, Nitric oxide, MK-801, Dependence, Oxidative stress

## Abstract

Morphine belongs to the class of opioids and is known for its potential to cause dependence and addiction, particularly with prolonged use. Due to the associated risks, caution must be taken when prescribing and limiting its clinical use. Overexpression of N-methyl-D-aspartate (NMDA) receptors, nitric oxide and cGMP pathway has been implicated in exacerbate the development of morphine dependence and withdrawal. Topiramate, an antiepileptic drug, interacts with various receptors, ion channels and certain enzymes. In this study, we investigated the effects of topiramate on morphine dependence in mice, specifically targeting NMDA/Nitric oxide/cGMP pathway. Mice were administered different doses of topiramate (intraperitoneally) during the development phase, 45 min prior to morphine administration. Topiramate (20 mg/kg) significantly reduced naloxone-induced withdrawal symptoms in morphine-dependent mice. Additionally, subeffective doses of topiramate, when co-administered with NMDA receptor antagonist MK-801 (0.05 mg/kg) or nitric oxide synthase inhibitors such as L-NAME (10 mg/kg, a non-specific NOS inhibitor) and 7-NI (20 mg/kg, a selective nNOS inhibitor), showed a marked reduction in withdrawal signs. However, the effect of topiramate (20 mg/kg) was abolished when co-administered with NMDA (75 mg/kg, an NMDA receptor agonist) or L-arginine (60 mg/kg, a NOS substrate). *Ex-vivo* analysis revealed that topiramate significantly reduced oxidative stress and downregulated the gene expression of nNOS, NR1, and NR2B in morphine-treated mice. Furthermore, the expression of NR1 and NR2B proteins in the hippocampus and cortex was significantly reduced in topiramate-pretreated mice. Hence, this finding suggest that topiramate mitigates morphine dependence and withdrawal by inhibiting oxidative stress and modulating the NMDA/NO pathway.

## Graphical abstract

Effect of topiramate on morphine dependence and underlying pathway. Topiramate negatively regulates NMDA receptor which in turns downregulates nitric oxide and oxidative stress and hence decreases morphine dependence and withdrawal.

**Figure:**
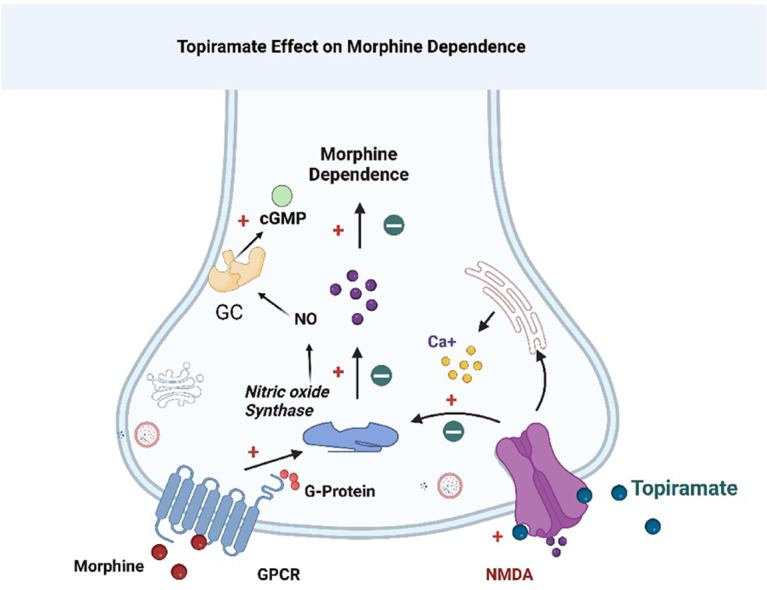

## 1 Introduction

Opioid use disorder is a chronic condition marked by persistent and relapsing behavior that significantly affects the Central Nervous System (CNS), particularly the brain and overall quality of life. Morphine, is a potent opioid analgesic often used in of chronic pain management can lead to dependence with prolong use. Morphine dependence is characterized by the development of withdrawal symptoms upon cessation or reduction of drug use. This condition is distinct from addiction, which involves compulsive drug seeking and use, despite its harmful consequences [1]. The withdrawal symptoms can be both physically and psychologically debilitating, including nausea, vomiting, diarrhea, muscle aches, respiratory depression, depression, and anxiety [[2], [3], [4]]. The central nervous system (CNS) is profoundly affected by morphine dependence, particularly the prefrontal cortex (PFC) and hippocampus. The PFC is crucial for regulating emotions, decision-making, and executive functions, all of which are compromised in substance dependence. The PFC play a pivotal role in the regulation of emotion and decision making, which are significantly affected by substance dependence like morphine [5]. The hippocampus, on the other hand, plays a vital role in memory formation and neuroplasticity, both of which contribute to the persistence of drug-related memories and cravings, which drive relapse. Withdrawal from morphine triggers a cascade of adverse effects due to the abrupt discontinuation of its influence on the CNS. For instance, it is reported that morphine withdrawal aversive memories significantly increase the level of H4K5ac and p-Brd4 in the hippocampus of morphine dependent rats, demonstrating the neurobiological changes associated with opioid dependence [6].

Morphine primarily exerts its effects by activating opioid receptors, which belong to a specific category of G protein-coupled receptors (GPCRs). These receptors are widely distributed throughout the central nervous system (CNS) and peripheral tissues [7]. Upon binding to these receptors, morphine triggers a cascade of intracellular signaling events that modulate various physiological and psychological processes. Morphine has a high affinity for μ-opioid receptors, which play a pivotal role in mediating its analgesic and euphoric effects. Upon activation, it modulates various intracellular signaling pathways including the opening of potassium channel, and the inhibition of calcium ion flux [8]. Such molecular interactions highlight the physiological and psychological effects of morphine use, which is critical in understanding the mechanism of morphine dependence.

N-methyl-D-aspartate (NMDA) receptors play a crucial role in the development of morphine tolerance and dependence. These receptors, a subtype of glutamate receptors, are essential for synaptic plasticity, memory function, and neuronal communication [9,10]. When glutamate, the primary excitatory neurotransmitter in the brain, binds to NMDA receptors, it facilitates the influx of calcium ions (Ca^2^⁺) into the neuron. This calcium influx triggers a cascade of intracellular signaling pathways, including the activation of protein kinases, production of nitric oxide (NO), and other secondary messengers, which collectively contribute to the strengthening of synaptic connections. This process, known as synaptic plasticity, is crucial for learning and memory but also underlies the development of drug tolerance and dependence [11]. In the context of morphine dependence, chronic exposure to morphine leads to increased activity and expression of NMDA receptors. Moreover, the NMDA receptor-mediated calcium influx and subsequent signaling pathways are implicated in the neuronal adaptations that underlie dependence and withdrawal symptoms. For instance, research has demonstrated that co-administration of NMDA receptor antagonists with morphine can prevent the development of tolerance and decrease withdrawal symptoms in both clinical and preclinical settings [12,13]. Furthermore, endogenous modulators of NMDA receptors, such as magnesium and zinc, have also been investigated for their potential to attenuate morphine tolerance and dependence [14,15]. Hence, NMDA receptors are integral to the mechanisms of morphine tolerance and dependence.

Nitric oxide (NO) is a versatile signaling molecule involved in various physiological and pathological processes including morphine dependence [16]. It is formed from L-arginine by Nitric oxide synthase (NOS) which exist in three main isoforms: neuronal NOS (nNOS), endothelial NOS (eNOS), and inducible NOS (iNOS). Each isoform plays a distinct role in various cellular functions. The nNOS is predominantly found in neurons and promote neuroplasticity, neurotransmission release, and are associated with neural pathways including in morphine tolerance and dependence [17,18]. Various studies have demonstrated that NOS inhibitors such as NG-nitroarginine methyl ester (L-NAME, nonspecific NOS inhibitor) and 7-Nitroindazole (7-NI, selective nNOS inhibitor) have shown to mitigate the neuroadaptive changes associated with morphine dependence [19]. Furthermore, Experimental findings suggest that co-administration of NOS inhibitors with morphine significantly reduces the severity of withdrawal signs, such as jumping, grooming, and weight loss in animal models [16]. It has been shown that NMDA receptor activation increases the level of NO, which may exacerbate the development of morphine tolerance and dependence [20]. Thus, these findings suggest involvement of NO in morphine dependence underscores the potential of targeting the NO signaling pathway as a therapeutic strategy.

Cyclic guanosine monophosphate (cGMP) is a secondary messenger with a variety of physiological processes and has been implicated in the modulating synaptic plasticity and neurotoxicity associated with morphine dependence [21]. It also regulates ion channels and gene expression [22]. Normally, it is catalyzed by guanylate cyclase (GC) which in turn is activated by nitric oxide. It has been reported by Javadi et al. that pioglitazone increases the level of cGMP in morphine rendered mice and hence potentiation the withdrawal symptoms [23].

Morphine dependence is also linked with oxidative stress and neuroinflammation in mesolimbic dopamine system and other addiction neurocircuitry [24]. Prolong morphine consumption causes elevated reactive oxygen species (ROS) thereby challenging the antioxidant defense system, microglial activation and neuroinflammation in reward pathway [24]. Studies have shown that prolonged morphine exposure reduces levels of crucial antioxidants such as glutathione (GSH) and catalase, while increasing lipid peroxidation (LPO) markers, indicating enhanced oxidative stress [25]. Excessive ROS accumulation damages proteins, lipids, and nucleic acids, and triggers pro-apoptotic signaling cascades implicated in morphine's adverse effects. Moreover, oxidative stress overload drives further neuroinflammation via positive feedback, establishing a self-perpetuating cycle that exacerbates morphine neurotoxicity and motivates continued drug self-administration [26]. Antioxidants have demonstrated efficacy in mitigating morphine-induced oxidative damage. Compounds like N-acetylcysteine have been shown to restore antioxidant levels, reduce ROS, and ameliorate neuroinflammatory responses. By counteracting oxidative stress, antioxidants can potentially attenuate the neurotoxic effects of chronic morphine use, offering a therapeutic avenue for managing dependence.

Topiramate, an antiepileptic drug, has shown promise as a therapeutic agent due to its multifaceted pharmacological properties. It modulates NMDA receptors, nitric oxide pathways and reduce oxidative stress, which are all implicated in the neurobiology of morphine dependence [27]. Central to its ability to antagonize NMDA receptor activity, topiramate can reduce calcium influx and subsequent neuronal excitability, which are critical in the pathophysiology of dependence [28]. Additionally, topiramate, inhibits nitric oxide pathway and oxidative stress which is often dysregulated in morphine dependence [29,30]. Furthermore, topiramate influences the nitric oxide signaling pathway by inhibiting nitric oxide synthase (NOS). This inhibition reduces NO production, thereby decreasing cGMP levels and modulating synaptic plasticity and neurotoxicity associated with morphine dependence. Hence, these effects suggest that topiramate might have the potential to treat morphine dependence with possible regulation of this pathway.

This study is the first comprehensive investigation into the effects of topiramate on morphine dependence, specifically focusing on its modulation of oxidative stress, NMDA receptor activity, and nitric oxide signaling pathways.

## 2 Materials and methods

### 2.1 Drugs and reagents

The following chemicals and reagents were utilized, each sourced from reputable suppliers: Morphine Sulfate: Sigma-Aldrich, Naloxone: Pfizer, Topiramate: Siam Pharmaceuticals, Islamabad, 7-Nitroindazole (7NI) and NG-Nitro-L-arginine methyl ester (L-NAME): Sigma-Aldrich, St. Louis, MO, USA, Aminoguanidine: Santa Cruz Biotechnology, NMDA Receptor Antagonist (MK-801): Merck, L-Arginine: Thermo Fisher Scientific, NMDA (Agonist of NMDA Receptor): Abcam, Antioxidant ELISA Kit: Elabscience, cAMP and cGMP Enzyme Immunoassay (EIA) System Kits: Thermo Fisher Scientific, Primers for Gene Expression Analysis: Qiagen, Griess Reagent: Enzo Life Sciences.

### 2.2 Experimental animals and housing conditions

In this study, NMRI mice in the 30 ± 2 g range purchased from NIH, Islamabad, Pakistan were used throughout the experiments. The animals were housed in an environment with regulated temperature, lighting cycles, and housing conditions. Mice were kept in standard laboratory cages with 4–5 mice per cage, in quarters that maintained a 12-h light-dark cycle, a constant room temperature of 25 °C, and unrestricted access to food and water. To minimize any extraneous factors that could affect study results, mice were acclimated to the laboratory environment 2 h prior to each experiment. In each experimental group there were 8–9 mice. All experiments adhered to the National Institutes of Health (NIH) guidelines for the ethical treatment and use of laboratory animals. The experimental protocols received prior approval from the Ethics Committee of Khyber Medical University in Peshawar, Khyber Pakhtunkhwa, Pakistan (Reference No. DR/KMU-AS&RB/RT/001437), as well as the Pak Austria Fachhochschule: Institute of Applied Sciences and Technology in Haripur, KPK, Pakistan (Reference No. PAF-IAST2023/07). Stringent ethical standards were upheld throughout the research process to ensure the humane and responsible conduct of studies involving animals.

### 2.3 Induction of morphine dependence in mice

To establish morphine dependence in the animals, a escalating dosage regimen of morphine sulfate was administered via intraperitoneal (i.p.) injection over four consecutive days. On each day, the animals received 50 mg/kg at 8 a.m., 50 mg/kg at 11 a.m., and a higher dose of 75 mg/kg at 4 p.m. The elevated final daily dose aimed to mitigate potential overnight withdrawal symptoms. On the fifth day, a single high dose of 100 mg/kg morphine sulfate was administered, followed 1 h later by an i.p. injection of naloxone at 4 mg/kg to precipitate opioid withdrawal and enable the assessment of dependence [16]. It is pertinent to mention that no mortality was observed at these recommended doses. The animals were closely monitored for at least 1 h following naloxone administration to evaluate the manifestation of opioid withdrawal symptoms. For this observation period, each animal was placed individually in a transparent plexiglass cylinder (dimensions: 40 cm × 25 cm x 45 cm) to facilitate video recording. Withdrawal signs meticulously documented included jumping behavior, diarrhea, excessive grooming, and weight changes. Body weight measurements were taken immediately before and after naloxone injection to quantify any weight loss associated with the precipitated withdrawal state.

To investigate the effect of topiramate and or coadministration of inhibitors on morphine dependence, different doses of topiramate (5, 10, 15, 20- mg/kg, i.p.) were administered alone and/or with inhibitors 45 min and 30 min respectively before each dose of morphine for five days consequently. The withdrawal signs were observed for 1 h after the last dose of 100 mg/kg of morphine (i.p.) followed by 4 mg/kg of naloxone (i.p.) on the fifth day by the experimentation. All the doses were selected based on our pilot study or previously published reports.

### 2.4 Experimental design and treatment

The behavioral experiment protocol lasted for five days, during which the mice were divided into different groups (n = 8, per group): saline, topiramate, morphine-dependent, and morphine with topiramate treatment. Topiramate was injected at doses of 5. 10, 15, and 20 mg/kg according to our pilot study. The different group received a daily dose of either saline, topiramate and/or morphine or a combination with the different Nitric Oxide Synthase and NMDA Inhibitors/Precursors. All treated drugs were dissolved in normal saline except 7NI (in 1 % DMSO).

The extracted brain of various groups was processed for antioxidant, PCR, and immunohistochemistry. The histopathological cohort's brain tissue was first preserved in 4 % paraformaldehyde, embedded into paraffin blocks, and trimmed into 4 μm sections using a microtome.

**Fig. 1:**
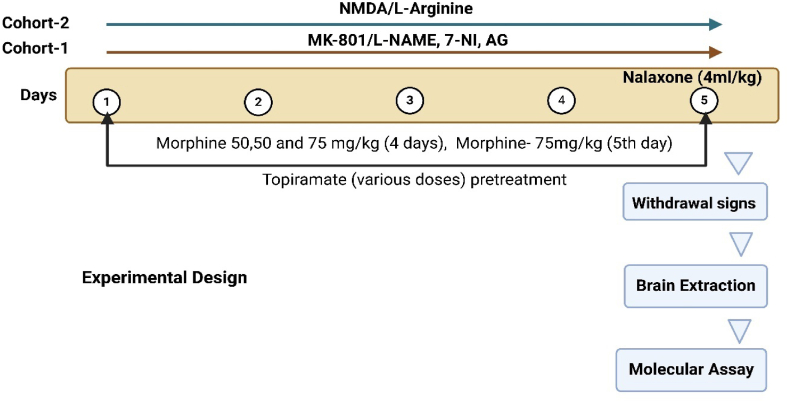
Experimental design and various treatment groups.

In all treated groups, morphine dependence was induced by administering morphine followed by either Topiramate or saline treatment for five consecutive days as shown in Fig. 1.

### 2.5 Histopathological assessment of brain tissue

The collected brain tissue samples were fixed in 4 % paraformaldehyde and processed for histological examination by decalcifying with nitric acid. Subsequently, the histological samples were embedded in paraffin blocks and sectioned into 4 μm thin slices using a microtome, following the protocol described by Alvi et al. [31]. Subsequent analysis was performed on the brain tissue sections.

### 2.6 Determination and analysis of oxidative stress biomarkers

The isolated cortical and hippocampal tissues underwent homogenization, followed by centrifugation at 1500 rpm for 30 min to separate the supernatant. The levels of glutathione (GSH), glutathione-S-transferase (GST), catalase, and lipid peroxidation (LPO) were assessed in the supernatant fractions. GSH content was quantified through the oxidation of GSH by DTNP, forming the yellow product 2-nitro-5-thiobenzoic acid, with absorbance measured at 412 nm using a microplate reader. GSH levels are expressed as moles/mg protein. GST activity was determined by measuring the absorbance at 340 nm of the CDNB conjugate product, with the extinction

coefficient used to calculate GST activity in moles CDNB conjugate/minute/mg protein. Catalase activity, reflecting the decomposition of H₂O₂, was evaluated by measuring absorbance at 340 nm and expressed as mol H₂O₂/min/mg protein [32]. Lipid peroxidation (LPO) was assessed by quantifying the terminal by-product malondialdehyde (MDA), with absorbance measured at 532 nm using a microplate reader. LPO levels are reported as nmol of TBARS per minute per milligram of protein [31].

### 2.7 Methodology and validation of genes expression analysis by qRT-PCR

Hippocampal and cortical tissues were collected from mice to analyze NR1, NR2B, and NOS gene expression. Total RNA was extracted using the RiboEX Total RNA Extraction Kit (GeneAll Biotechnology, Seoul, Korea), and its quality was verified by A260/A280 ratio. cDNA was synthesized from RNA using the First Strand cDNA Synthesis Kit (Thermo Fisher Scientific). qRT-PCR reactions were performed in a 20 μL volume with SYBR Green, and thermal cycling conditions included initial denaturation at 95 °C for 15 min, followed by 40 cycles of 95 °C for 10 s and 60 °C for 1 min. Melting curve analysis validated primer specificity. Gene expression was normalized to the beta-2-microglobulin (B2M) housekeeping gene, and relative expression was calculated using the ΔCT method. All experiments were performed in triplicate, with strict quality control throughout to ensure reliable results [16].

### 2.8 Immunohistochemistry (IHC) of hippocampus and cortex

Immunohistochemical staining of brain tissue sections was performed to analyze the expression of NR2A and NR2B, in accordance to our previously described protocol [33]. The tissue sections mounted on slides underwent deparaffinization using three successive changes of xylene, followed by rehydration through a graded series of ethanol solutions (100 %–70 %). The slides were then immersed in 0.01M phosphate-buffered saline (PBS) for 10 min and rinsed with distilled water. After antigen retrieval, the tissue sections were incubated overnight with primary antibodies against NR2A and NR2B. Subsequently, the slides were incubated for 2 h with appropriate biotinylated secondary antibodies, followed by a 1-h incubation with avidin-biotin complex (ABC) reagents (Standard Vectastain ABC Elite Kit; Vector Laboratories, Burlingame, CA, USA) at optimal room temperature. The sections were rinsed with PBS and then stained with 3,3-diaminobenzidine (DAB) solution. To prepare the slides for microscopic evaluation, the tissue sections were dehydrated through a graded series of ethanol solutions (70 %, 95 %, and 100 %), cleared in xylene, and mounted with a coverslip using a compatible mounting medium. The slides were allowed to air-dry before analysis. The immunohistochemically stained sections were evaluated using a light microscope (Olympus, Japan) connected to a high-quality digital photomicrography system. Digital images of the tissue sections were acquired at multiple fields (5 images per slide) using the light microscope. Quantitative analysis of NR1 and NR2 antibody staining was performed using ImageJ software [34].

### 2.9 Statistical analysis

All statistical analyses were conducted using GraphPad Prism 8 software. Data were evaluated using one-way analysis of variance (ANOVA), followed by Tukey’s post hoc test for multiple comparisons. Morphological data were quantified using ImageJ software. Statistical significance was defined as *p* < 0.05. Symbols such as ∗ or # indicate *p* < 0.05, ∗∗ or ## indicate *p* < 0.01, and ∗∗∗ or ### indicate *p* < 0.001. All results are presented as mean ± standard error of the mean (SEM).

## 3 Results

### 3.1 *In vivo* behavior study

The result of the behavioral study of morphine withdrawal in various treated groups are listed below.

#### 3.1.1 Dose response effect of topiramate on morphine withdrawal signs

**Fig. 2:**
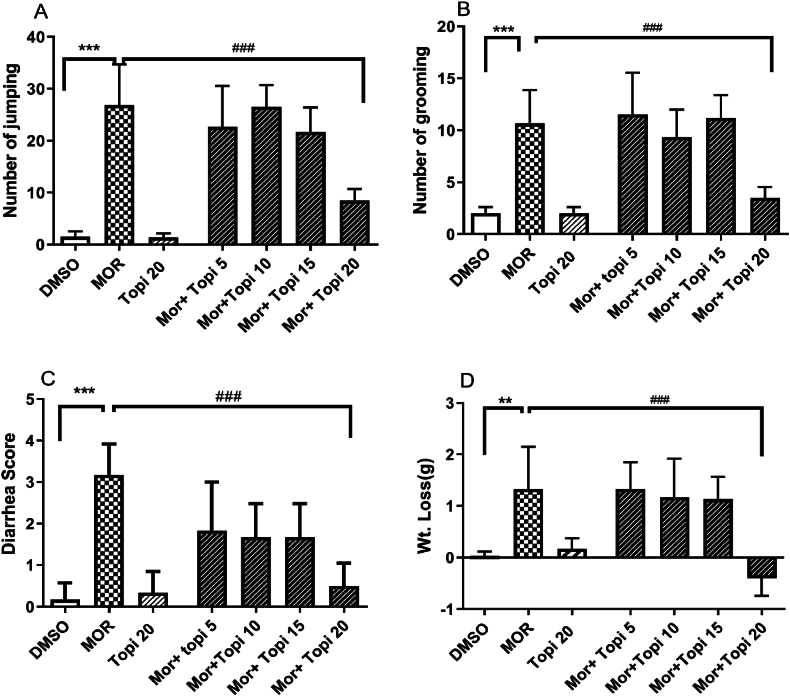
**(A**–**D).** Effect of different doses of topiramate (Topi) on morphine (MOR) dependence and withdrawal, administered (i.p.) 45 min before each dose of MOR administration for 5 days. Data are expressed as mean ± S.E.M. for withdrawal signs such as jumping, Number of grooming, Diarrhea and Weight loss after naloxone injection for 8–9 mice. ^*###*^*p* < 0.001 for Top 20 mg/kg compared to morphine treated group. *∗∗p* < 0.01, *∗∗∗p* < 0.001 for morphine compared to control/vehicle treated group.

Fig. 2 (A-D) shows the dose-dependent effect of topiramate on naloxone-precipitated withdrawal symptoms in morphine-dependent mice. Topiramate (5 mg/kg to 20 mg/kg, i.p.) was administered 45 min prior to morphine injections for 5 days. Morphine induced significant withdrawal symptoms compared to the control (∗∗∗*p* < 0.001, Fig. 2 A-D). At 20 mg/kg, topiramate significantly reduced withdrawal behaviors, including jumping (^###^*p* < 0.001, A), grooming (^###^*p* < 0.001, Fig. 2 B), weight loss (^###^*p* < 0.001, Fig. 2 C) and diarrhea (^##^*p* < 0.01, Fig. 2 D). However, on Day 5, the 20 mg/kg dose did not significantly differ from the vehicle control (*p* > 0.05, A-D).

#### 3.1.2 Effect of non-selective nitric oxide synthase (NOS) inhibitor on topiramate activity in morphine dependent mice

**Fig. 3:**
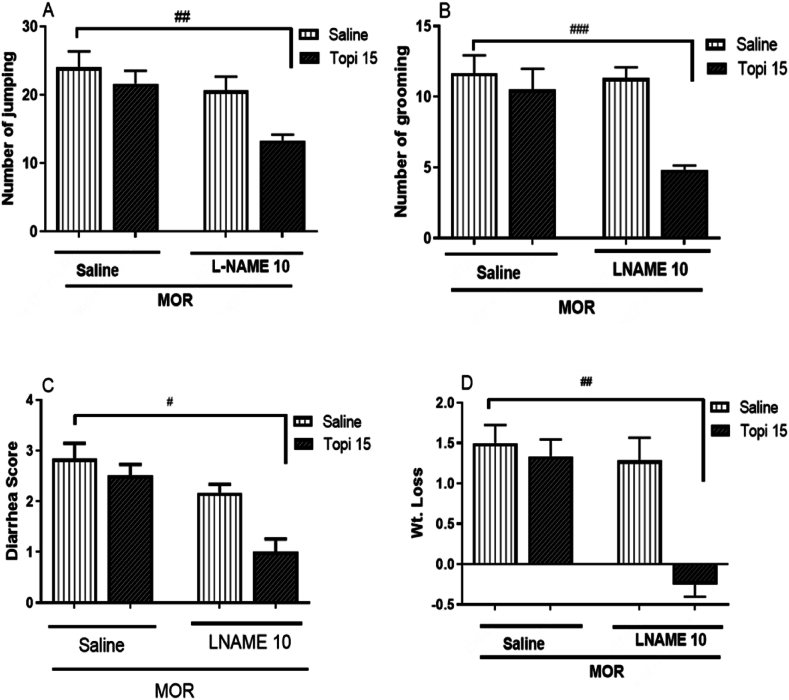
**(A**–**D).** Effect of Topiramate on naloxone-induced withdrawal signs in morphine-dependent mice. Topiramate (Topi) (15 mg/kg) was administered 45 min while L-NAME (10 mg/kg) was administered 30 min before each dose of MOR injection for 5 days. Data are presented as mean ± S.E.M for withdrawal signs of 8–9 mice. A: Number of jumping after naloxone injection. B: Number of grooming. C: Diarrhea. D: Weight loss after naloxone injection. ^#^*p* < 0.05, ^##^*p* < 0.01, and ^###^*p* < 0.001 for Topiramate (10 mg/kg) compared to the morphine treated group.

Fig. 3 (A-D) shows the effect of topiramate (15 mg/kg) alone and in combination with L-NAME (10 mg/kg, i.p.) on morphine withdrawal signs. L-NAME alone did not significantly affect withdrawal symptoms (*p* > 0.05 Fig. 3A-D), confirming its subeffective dose. However, combining topiramate with L-NAME significantly attenuated withdrawal symptoms, including reduced jumping (^##^*p* < 0.01, Fig. 3A), grooming (^###^*p* < 0.001, Fig. 3B), diarrhea severity (^#^*p* < 0.05, Fig. 3C), and weight loss (^##^*p* < 0.01 Fig. 3D), compared to the morphine-only group. These results highlight a synergistic interaction between topiramate and L-NAME in mitigating morphine withdrawal signs.

#### 3.1.3 Effect of 7-N1 on topiramate activity in morphine dependent mice

**Fig. 4:**
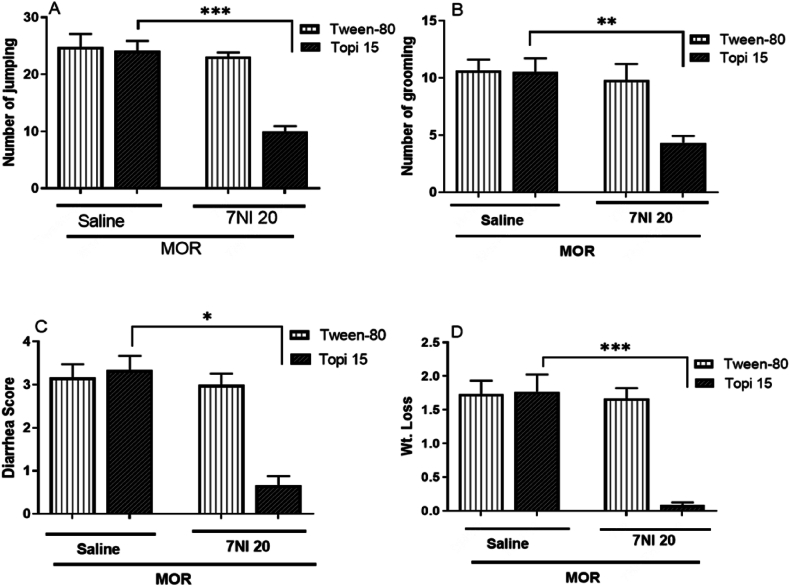
Effect of combined administration of topiramate (15 mg/kg) and 7-NI (20 mg/kg) on withdrawal symptoms in morphine-dependent mice. The results are expressed as mean ± S.E.M for withdrawal signs in 8–9 mice per group. No significant differences were observed. A: Number of jumps after naloxone administration ∗∗∗*p* > 0.05, B: Number of grooming episodes after naloxone ∗∗*p* > 0.05, C: Diarrhea score after naloxone ∗*p* > 0.05, and D: Weight loss after naloxone ∗∗∗*p* > 0.05 for the group treated with topiramate (10 mg/kg) + 7-NI compared to the morphine-only group.

Fig. 4 (A-D), demonstrates the effect of co-administering topiramate with 7-NI, a selective nNOS inhibitor, on morphine withdrawal signs. While 7-NI alone (20 mg/kg) had no significant effect (*p* > 0.05), combining it with topiramate significantly reduced withdrawal symptoms, including jumping (∗∗∗*p* < 0.001, Fig. 4. A), grooming (∗∗*p* < 0.01, Fig. 4B), diarrhea (∗*p* < 0.05, Fig. 4C), and weight loss (∗∗∗*p* < 0.001, Fig. 4D) compared to the morphine-only group. These results suggest a synergistic interaction between topiramate and 7-NI in mitigating withdrawal signs, highlighting the role of nNOS in the neuroprotective effects of topiramate.

#### 3.1.4 Effect of iNOS inhibitor aminoguanidine on topiramate activity in morphine dependent mice

**Fig. 5:**
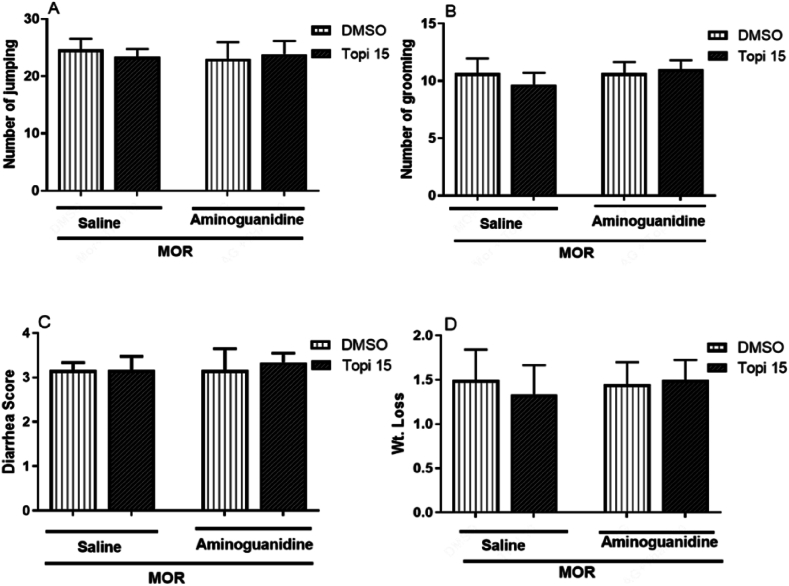
(A–D)Effect of co-administration of topiramate (Top) with Aminoguanidine (50 mg/kg) on morphine (MOR) withdrawal signs. Data are presented as mean ± S.E.M for withdrawal syndrome of 8–9 mice. A: Number of jumping after. B: Number of grooming. C: Diarrhea and D: Weight loss (g) after naloxone injection. ^#^*p* < 0.05, ^##^*p* < 0.01 for Topiramate 10 mg/kg + Aminoguanidine compared to morphine-treated group.

Fig. 5 (A-D), shows the effect of aminoguanidine, an iNOS and cGMP inhibitor, on topiramate activity in morphine-dependent mice. Co-administration of aminoguanidine (50 mg/kg) with a subeffective dose of topiramate (15 mg/kg) did not significantly alter naloxone-induced withdrawal signs, including jumping, grooming, diarrhea, and weight loss (*p* > 0.05, Fig. 5A-D). These findings suggest that the effect of topiramate is not due to modulation of iNOS and cGMP reduction, suggesting that the protective effect of topiramate is not due to inhibition of iNOS and cGMP.

#### 3.1.5 Effect of L-Arginine on topiramate activity in morphine dependent mice

**Fig. 6:**
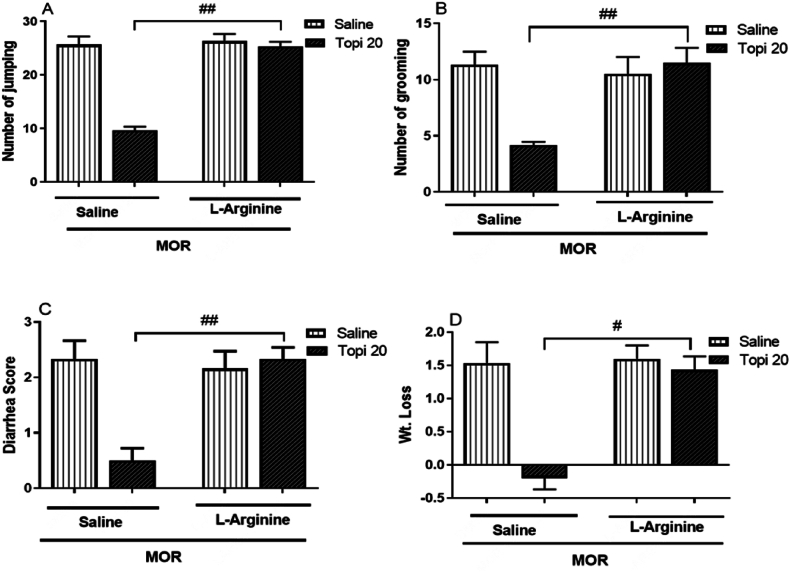
**(A**–**D).** Effect of co-administration of topiramate (Topi) with L-arginine (60 mg/kg) on topiramate dependence and withdrawal symptoms. Topiramate and L-arginine were administered 45 min and 30 min before each dose of topiramate injection for 5 consecutive days. The data are presented as mean ± S.E.M. for withdrawal signs in 8–9 mice. A: Number of jumping. B: Number of grooming. C: Diarrhea. D: Weight loss following naloxone injection. Statistical analysis revealed significant differences: ^*#*^*p* < 0.05, ^*##*^*p* < 0.01, for the Topi (15 mg/kg) + L-arginine group compared to the topiramate-treated morphine group.

Fig. 6(A–D), demonstrates the effect of co-administering L-arginine (60 mg/kg) with topiramate (20 mg/kg) on naloxone-induced morphine withdrawal signs. L-arginine significantly attenuated the protective effects of topiramate by reversing its impact on withdrawal symptoms, including jumping (^##^*p* < 0.01, Fig. 6A), grooming (^##^*p* < 0.01, Fig. 6B), diarrhea (^##^*p* < 0.01, Fig. 6C), and weight loss (^#^*p* < 0.05, Fig. 6D). L-arginine alone did not affect withdrawal symptoms (^#^*p* > 0.05, Fig. 6A-D), confirming its subeffective dose. These findings provide valuable insights into the interplay between topiramate and nitric oxide in the context of naloxone-induced withdrawal syndrome, highlighting the potential mechanisms underlying the protective effects of topiramate due to nitric oxide inhibition.

#### 3.1.6 Effect of NMDA receptor antagonist on topiramate activity in morphine dependent mice

**Fig. 7:**
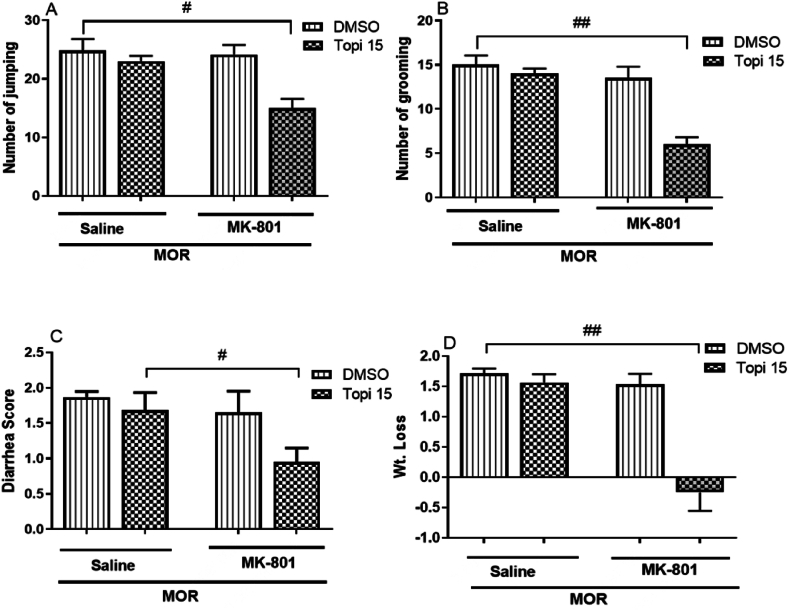
**(A**–**D).** Shows the effect of co-administration of topiramate (Topi, 15 mg/kg) with MK-801 (0.05 mg/kg) on naloxone induced withdrawal symptoms. Topiramate and MK-801 were administered 45 min and 30 min before each dose of morphine (MOR) injection for 5 consecutive days. The data are presented as mean ± S.E.M. for withdrawal signs in 8–9 mice. A: Number of jumping. B: Number of grooming. C: Diarrhea. D: Weight loss following naloxone injection. Statistical analysis revealed significant differences: ^*#*^*p* < 0.5, ^*##*^*p* < 0.01, for the Topi (15 mg/kg) + MK-801 group compared to the topiramate-treated group.

Fig. 7(A–D), illustrates the effect of co-administering the NMDA receptor antagonist (MK-801, 0.05 mg/kg) with a subeffective dose of topiramate (15 mg/kg) on naloxone-induced morphine withdrawal symptoms. The combination significantly enhanced topiramate effects, reducing jumping (^#^*p* < 0.05, Fig. 7A), grooming (^##^*p* < 0.01, Fig. 7B), diarrhea (^#^*p* < 0.05, Fig. 7C), and weight loss (^##^*p* < 0.01, Fig. 7D), compared to topiramate or MK-801 alone, which had no significant effects. These results suggest that NMDA receptor antagonism potentiates the effects of topiramate in alleviating morphine withdrawal symptoms.

#### 3.1.7 Effect of NMDA receptor agonist on topiramate activity in morphine dependent mice

**Fig. 8:**
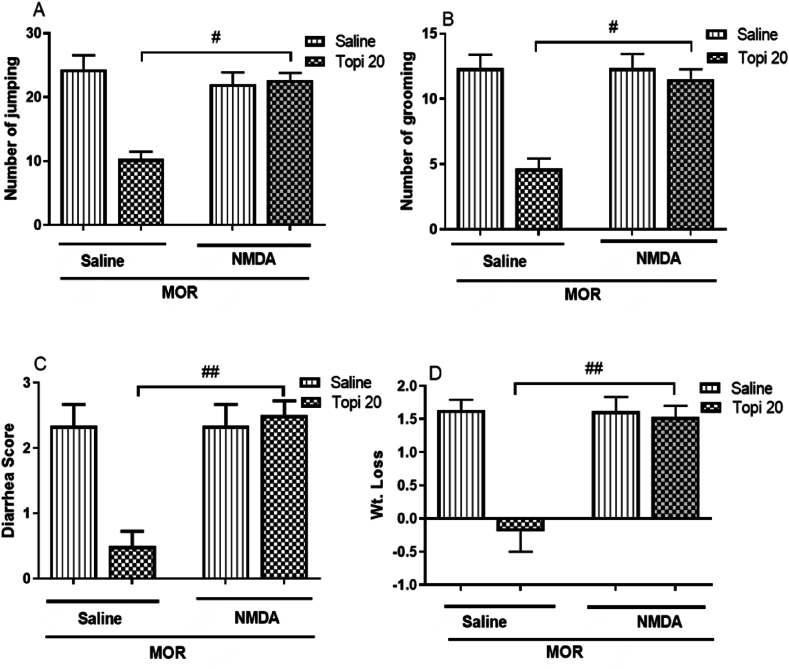
Effect of co-treatment of topiramate (Topi, 20 mg/kg) with NMDA (0.05 mg/kg) on naloxone induced withdrawal symptoms. Topiramate and MK-801 were administered 45 min and 30 min before each dose of morphine (MOR) injection for 5 consecutive days. The data are presented as mean ± S.E.M. for withdrawal signs in 8–9 mice. A: Number of jumping. B: Number of grooming. C: Diarrhea. D: Weight loss following naloxone injection. Statistical analysis revealed significant differences: ^#^*p* < 0.5, ^##^*p* < 0.01, for the Topi (20 mg/kg) + NMDA group compared to the morphine-treated group.

Fig. 8(A–D) shows the effect of co-administering an NMDA receptor agonist (0.05 mg/kg) with topiramate (20 mg/kg) on naloxone-induced morphine withdrawal symptoms. Co-administration significantly reduced the protective effects of topiramate on withdrawal signs, including jumping (^#^*p* < 0.05, Fig. 8A), grooming (^#^*p* < 0.05, Fig. 8B), diarrhea (^##^*p* < 0.01, Fig. 8C), and weight loss (^##^*p* < 0.01, Fig. 8D). The NMDA agonist alone had no significant effect (*p* > 0.5, Fig. 8A-D). These findings confirm that topiramate effect on morphine withdrawal is mediated through the inhibition of NMDA receptors.

### 3.2 Molecular *Ex-vivo* study

#### 3.2.1 Effect of topiramate on oxidative stress markers

**Fig. 9:**
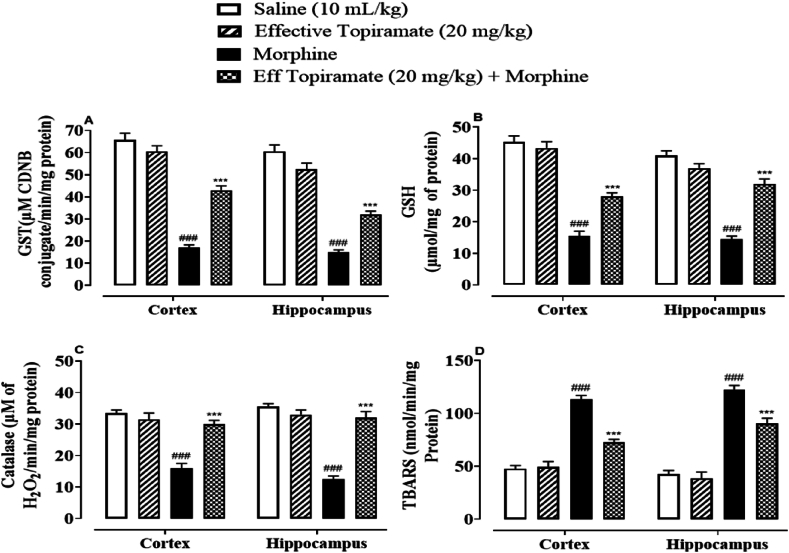
(A–D). Graphical representation of the effect of Topiramate against (A) glutathione-S-transferase (GST), (B) reduced glutathione (GSH), (C) catalase and (D) lipid peroxidation (LPO) in morphine treated mice cortex and hippocampal tissues. Values are expressed as mean ± SEM (n = 7/group). One-way ANOVA with post-hoc Tukey’s test. ^###^*p* < 0.001 denotes a significant difference from the saline group, only effective topiramate treatment group has no significant difference vs. Saline group. ∗∗∗*p* < 0.001 denotes a significant difference from the disease group (Morphine).

In order to determine the involvement of oxidative stress in morphine withdrawal symptoms, various oxidative stress parameters were studied in hippocampus and cortex. In the cortical and hippocampus tissues of morphine-treated mice, GST, GSH and catalase activity were markedly decreased, although LPO was elevated (^###^*p* < 0.001 vs. Saline group, Fig. 9 A-D). Topiramate significantly decreased LPO while restoring GST, GSH and catalase in the treated groups. The activity of GST, GSH and catalase were noticeably reduced in morphine-treated cortex and hippocampal tissues, although LPO was increased. Topiramate dramatically reduced LPO while restoring GST, GSH and catalase in the treated groups (∗∗∗*p* < 0.001 vs. Morphine group, Fig. 9 A-D).

### 3.3 Quantification of mRNA levels in cortex and hippocampus

#### 3.3.1 Quantification of mRNA levels nNOS, NR1, NR2B and iNOS

**Fig. 10:**
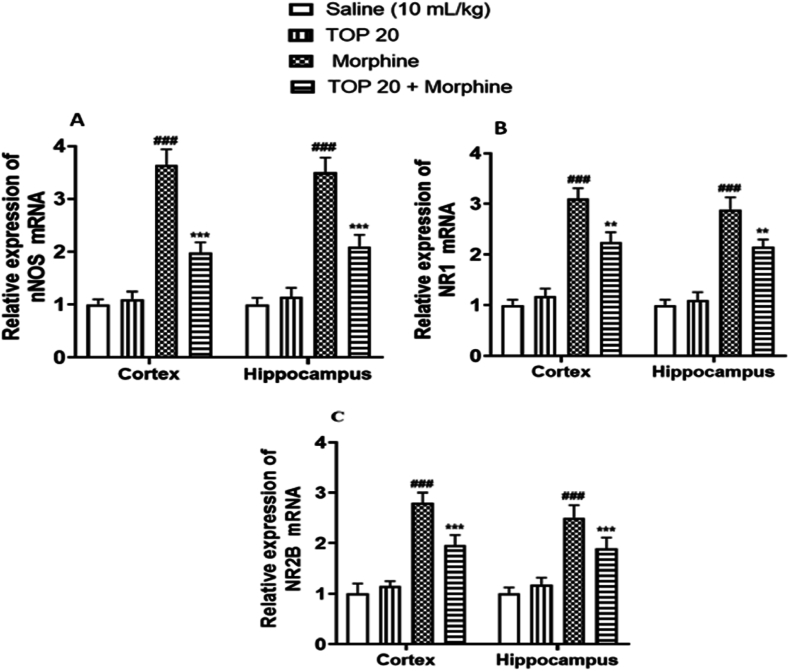
**(A**–**C).** Inhibitory effect of Topiramate against mRNA of (A) nNOS, (B) NR1 and (C) NR2B expression in morphine treated mice cortex and hippocampal tissues using real-time polymerase chain reaction (RT-PCR) technique. Values expressed as mean ± SEM (n = 8/group). One-way ANOVA with post-hoc Tukey’s test. ^**###**^*p* < 0.001 denotes a significant difference from the saline group, only effective topiramate treatment group has no significant difference vs. Saline group. ∗∗*p* < 0.01 and ∗∗∗*p* < 0.001 denotes a significant difference from the disease group (Morphine).

Through RT-PCR the fold expression of nNOS, NR1, NR2B and iNOS in morphine treated mice cortex and hippocampal tissues were determined. The Morphine administered group indicates increased expression of nNOS, NR1 and NR2B mRNA levels (^###^*p* < 0.001 vs. Saline group, Fig. 10A-C). However, topiramate treatment caused a significant decrease in expression levels of nNOS, NR1 and NR2B as compared to disease group (∗∗*p* < 0.01 and ∗∗∗*p* < 0.001 vs. Morphine group, Fig. 10 A-C).

#### 3.3.2 Effect of topiramate on iNOS in morphine treated group

**Fig. 11:**
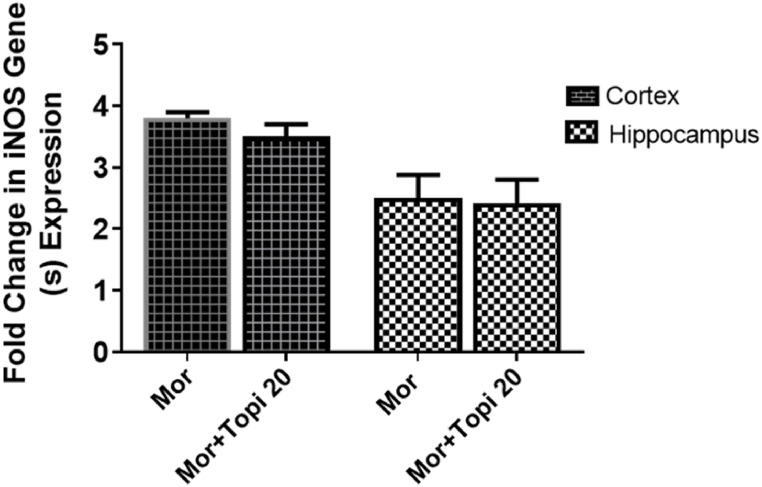
Expression of iNOS gene in Hippocampus and Cortex in morphine and topiramate treated group. No significance was found *p* > 0.05.

To study the effect of iNOS gene in the involvement of morphine dependence and the effect of topiramate on the expression of iNOS in morphine dependent mice, PCR analysis was performed (Fig. 11). No significant effect was observed in the expression of iNOS gene in hippocampus and cortex of morphine dependent and topiramate treated morphine dependent mice. This result also confirmed the behavior study where no such effect was observed when treated with aminoguanidine (Fig. 11, *p* > 0.05).

### 3.4 Effect of topiramate on morphine-induced NMDA-receptors expression

**Fig. 12:**
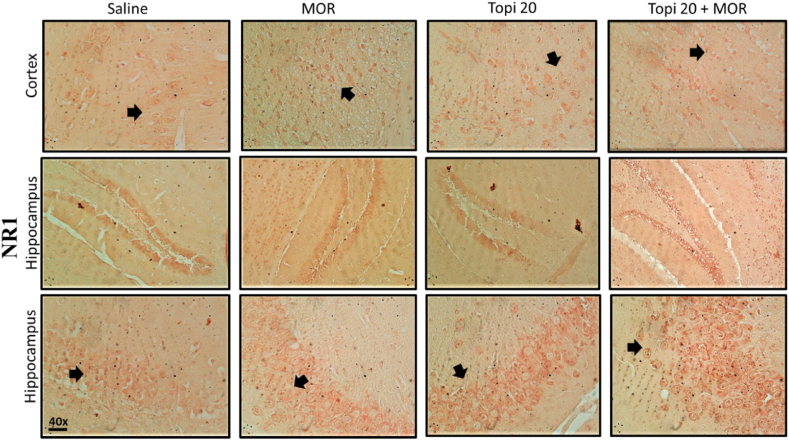
Effect of Topiramate treatment on morphine-induced NMDA-receptor expression using immunohistochemical analysis, showing the effects of NR1 in the mice cortex and hippocampal region. Scale bar 50 μm, magnification 40 × (n = 8/group).

**Fig. 13:**
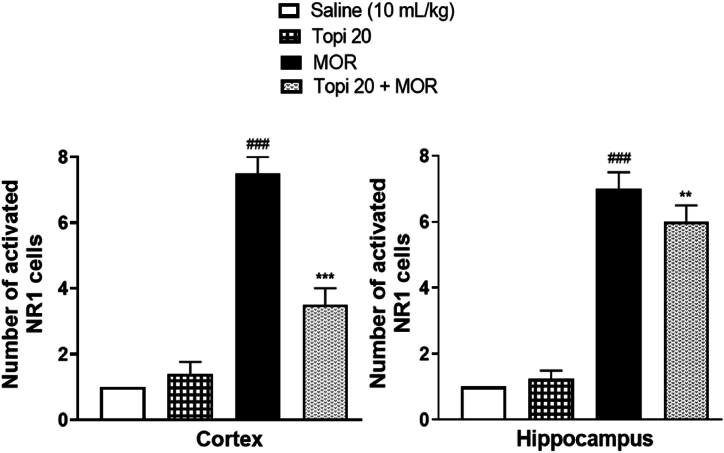
Inhibitory effect of Topiramate against NR1 expression in Morphine-treated mice cortex and hippocampal region, using the immunohistochemical technique. Values expressed as mean ± SEM (n = 8/group). One-way ANOVA with post-hoc Tukey’s test. ^**###**^*p* < 0.001 denotes a significant difference from the saline group, **∗∗***p* < 0.01, **∗∗∗***p* < 0.001 demonstrate a significant difference from the disease group (Morphine).

**Fig. 14:**
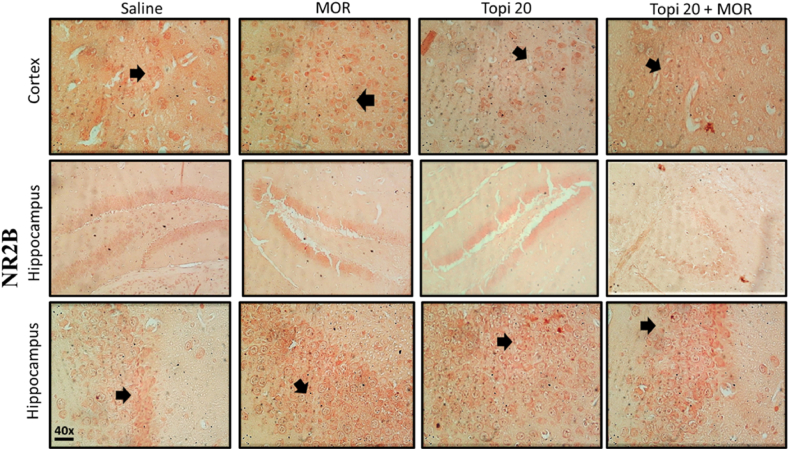
Effect of Topiramate treatment on morphine-induced NMDA-receptor expression using immunohistochemical analysis, showing the effects of NR2B in the mice cortex and hippocampal region. Scale bar 50 μm, magnification 40 × (n = 8/group).

**Fig. 15:**
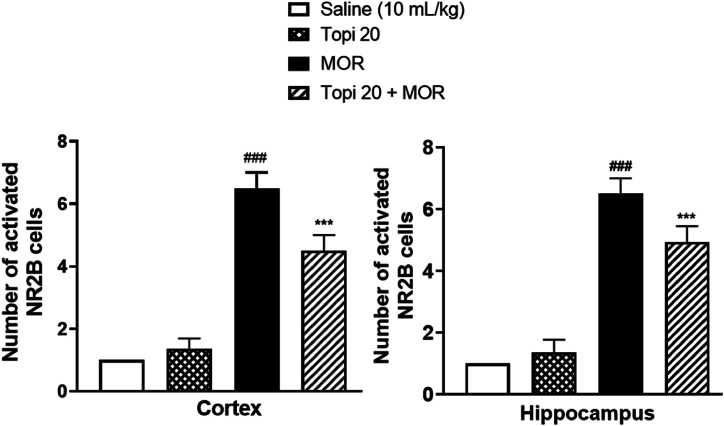
Inhibitory effect of Topiramate against NR2B expression in Morphine-treated mice cortex and hippocampal region, using the immunohistochemical technique. Values expressed as mean ± SEM (n = 8/group). One-way ANOVA with post-hoc Tukey’s test. ^**###**^*p* < 0.001 shows a significant difference when morphine treated group was compared to saline group. **∗∗∗***p* < 0.001 demonstrates a significant difference when drug treated morphine group was compared to disease group (only Morphine treated group).

To study the effects of Topiramate on the morphine-induced NMDA-receptor activation and expression in the cortical and hippocampal regions of mice brain, immunohistochemical analysis was performed for both NR1 and NR2B subunits of NMDA receptor. Our results clearly demonstrate the induction of both NR1 (Fig. 12) and NR2B (Fig. 14) by morphine in hippocampus and cortex brain regions after morphine-treatment (^###^*p* < 0.001 vs Saline group). However, our drug topiramate (20 mg/kg) treatment significantly modulated the expression of both subtypes of NMDA; NR1 and NR2B, hence presenting neuronal protection by the Topiramate in morphine-induced toxicity (Fig. 13, Fig. 15, ∗∗*p* < 0.01, ∗∗∗*p* < 0.001 and ∗∗∗*p* < 0.001 drug treated morphine group compared to only morphine group).

## 4 Discussion

Morphine dependence involves complex neurobiological mechanisms, including oxidative stress, NMDA receptor activity, nitric oxide (NO) production, and cGMP signaling. These pathways are interconnected and contribute to the development and maintenance of opioid dependence. The aim of the current study was to inves tigate the effects of topiramate on morphine dependence, focusing on modulation of the NMDA/NO/cGMP pathway and morphine induced oxidative stress. Morphine is an opioid analgesic that binds to the opioid receptor and regulates certain important functions. Morphine addiction is a chronic condition which is characterized by compulsive drug seeking and use behavior, even in spite of negative consequences. Morphine is currently used in clinical practice to overcome chronic pain associated with trauma or cancer and for recreational purposes. However, it rapidly develops dependency, whichever when stopped or decreased the dose of morphine induces withdrawal signs. This unique drawback limits its use for clinical purposes and poses a serious threat to public health in the form of drug addiction [35]. The major objective of the current study was to investigate the potential mechanism of morphine withdrawal effects in morphine dependent animal model and to study the effect of topiramate on morphine withdrawal, taking into consideration the activity of oxidative stress, NMDA, and nitric oxide pathway. Topiramate is an antiepileptic drug, which has been shown to modulate the activity of various neurotransmitters including NMDA and nitric oxide [29].

Oxidative stress is a condition in which there is an imbalance between the production of reactive oxygen species (ROS) and the ability of the body to detoxify it with the help of antioxidants production. Oxidative stress has been implicated in the development of a number of diseases, including cancer, heart disease, and neurodegenerative disorders [36,37]. Furthermore, the role of oxidative stress in opioid dependence has been highlighted in several studies. It has been reported that chronic morphine administration increases reactive oxygen species (ROS) and decreases antioxidant defenses, contributing to neurotoxicity and dependence [38,39]. Our study adds to this body of evidence by demonstrating that topiramate significantly reduces oxidative stress markers (e.g., LPO) while enhancing antioxidant levels (e.g., GST, GSH, and catalase) in morphine-dependent mice. These results align with the findings where the researcher showed that antioxidants mitigate morphine-induced oxidative damage [40]. In current study, Topiramate has been shown to have neuroprotective effects by reduce oxidative stress and increasing the level of antioxidants as shown in Table 1. A number of studies have examined the effects of topiramate on oxidative stress and morphine dependence. It has been reported that when rats were treated with chronic morphine for 14 days showed increased levels of ROS and decreased levels of antioxidants [41]. This study is in line with our current results shown in Fig. 10A-D, where topiramate significantly reduced the levels of ROS and increased profoundly the levels of antioxidants. Hence, it suggests that topiramate may protect against the oxidative stress that is associated with morphine dependence [42].

**Table 1:**
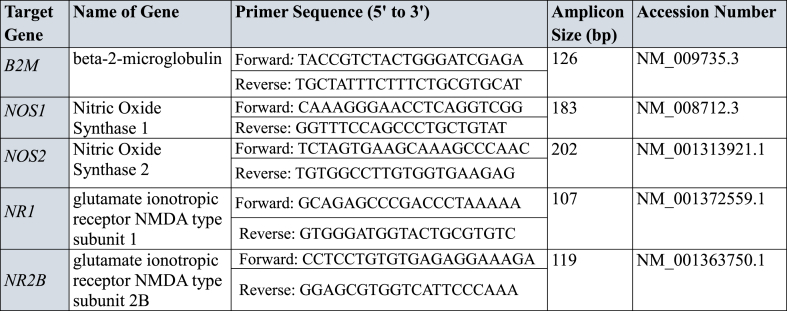
Genes Primers along with accession number.

Nitric Oxide (NO) is a gaseous signaling molecule which is involved in a variety of physiological and pathological processes including pain modulation, learning and memory, neuronal plasticity, and addiction. NO is produced by the enzyme nitric oxide synthase (NOS) within a biological system. There are three isoforms of NOS i.e. neuronal NOS (nNOS), endothelial NOS (eNOS) and inducible NOS (iNOS) [43]. nNOS and eNOS are constitutively expressed, while iNOS is only expressed in response to certain stimuli, such as inflammation, stress and certain type of trauma or drug insult. Morphine addiction is also thought to be a result from changes in neurotransmitters in the brain's reward circuitry, which is regulated by NO [44]. There have been a number of studies that have investigated the effects of topiramate on various disease model including drug dependence. A recent study found that topiramate inhibited the activity of nNOS in rat brain slices of depressed animals [45]. Another report found that topiramate reduced the expression of iNOS in rat brain slices that were exposed to morphine [46]. In addition, a clinical trial found that topiramate was effective in reducing the withdrawal symptoms associated with morphine dependence [47]. As shown in Fig. 3(A–D), our results also show that topiramate treatment significantly reduced the morphine withdrawal signs when coadministered with L-NAME. Topiramate selectively inhibited nNOS in the current study as evident from Fig. 4 Jumping (∗∗∗*p* > 0.001, Fig. 4 A), grooming (∗∗*p* > 0.01, Fig. 4 B), diarrhea (∗*p* > 0.05, Fig. 4 C), and weight loss (∗∗∗*p* > 0.001, Fig. 4 D). However, no significant changes were observed in withdrawal symptoms when the animals were treated with specific iNOS inhibitor. Similarly, the PCR results were also in line with the behavior studies. The cortex and hippocampus of the morphine treated mice significantly expressed the level of nNOS however, no significant change was found in the expression of iNOS. Hence, it is clear that topiramate significant effect on morphine dependence in mice is partly regulated by inhibition of nitric oxide especially by downregulating nNOS isoform of NOS.

Previous studies have established that the activation of the NMDA receptor by glutamate is a critical factor in the development of opioid tolerance and dependence [48]. For instance, various studies demonstrated that NMDA receptor antagonists, such as ketamine and memantine, effectively attenuate morphine tolerance and dependence [49,50]. Our findings corroborate these results by showing that topiramate, which has NMDA receptor antagonistic properties, similarly reduces morphine withdrawal symptoms and dependence. There is significant evidence that topiramate down regulates NR1 and NR2B activity of NMDA receptor and neuronal nitric oxide synthase. The NR1 and NR2B subunits play a key role in NMDA receptor activity and was found to increase morphine dependence and synaptic plasticity [51,52]. These studies are in line with our current study, where morphine administration significantly increased the expression of NR1 and NR2B in the hippocampus and cortex when treated for 5 days (Fig. 11, Fig. 13). However, concomitant administration of topiramate with morphine significantly decreased the expression of NR1 and NR2B activity of NMDA receptor along with significant reduction of morphine dependence. The results of the current study are consistent with the previously published reports which showed an important role of NMDA receptor in the development of morphine dependence [9,53]. Nitric oxide is a crucial biosignaling molecule, synthesized from L-arginine by the action of 3 different isoforms of nitric oxide synthase present in the brain and various other tissues [54]. It plays an important role in the development and expression of morphine dependence and withdrawal [16]. It has been reported that inhibition of NR1 and NR2B subunit of NMDA receptor reduces neuronal excitability and also synaptic plasticity. Both of these two mechanisms have a key role in the development and expression of morphine dependence and tolerance. More studies are needed to determine the benefit of use of topiramate in morphine dependence, as it has an important side effect of cognitive impairment. Further, the current results added significantly to a growing body of literature suggesting that glutamate-mediated neurotransmission via NMDA receptors plays a significant role in opioid dependence. Also, activation of this receptor also leads to activation of induction of nNOS. Thus, therapeutic strategies aimed at modulating oxidative stress, glutamatergic activity, and nitric oxide pathway, by topiramate, might have substantial potential in treating morphine dependence and withdrawal.

In comparison to other NMDA antagonists and antioxidants, topiramate offers unique advantages by simultaneously targeting multiple pathways involved in morphine dependence. This multifaceted action may contribute to its superior efficacy in reducing morphine withdrawal symptoms. Moreover, while antioxidants such as N-acetylcysteine specifically address oxidative stress, they do not modulate NMDA receptor activity. Topiramate ability to influence both NMDA receptor-mediated excitotoxicity and oxidative stress presents a comprehensive therapeutic strategy for managing opioid dependence. This dual mechanism of action highlights the potential of topiramate as a more effective treatment option compared to agents targeting a single pathway.

## 5 Conclusion

In summary, our study supports the therapeutic potential of topiramate in mitigating morphine dependence through its combined effects on the NMDA and NO signaling pathway and oxidative stress. Compared to the findings of the existing literature, we highlighted the unique benefits of topiramate in the treatment of opioid dependence through a multifaceted approach.

### 5.1 Limitations of the current study

Despite the significant findings of our study, several limitations should be acknowledged. Firstly, the study was conducted using animal model, and the results may not fully translate to humans due to species-specific differences in drug metabolism and neural responses. Secondly, the mechanisms underlying topiramate modulation of oxidative stress and NMDA/NO pathways were based on existing literature and our experimental data, but further molecular studies are needed to elucidate these pathways in greater detail with associated biomarkers other than the current biosignaling. Addressing these limitations in future studies will help to validate and extend our findings, ultimately contributing to more effective therapeutic strategies for opioid dependence.

## CRediT authorship contribution statement

**Shabir Hussain:** Writing – original draft, Validation, Investigation. **Haji Bahadar:** Writing – review & editing, Methodology, Investigation, Formal analysis, Conceptualization. **Muhammad Imran Khan:** Writing – original draft, Resources, Conceptualization. **Neelum Gul Qazi:** Writing – review & editing, Investigation, Formal analysis, Data curation. **Shabnum Gul Wazir:** Validation, Investigation, Formal analysis. **Habab Ali Ahmad:** Writing – original draft, Investigation, Data curation.

## Data availability statement

All the data supporting the findings of this study are contained within the manuscript. No additional data were generated or analyzed during the current study which can be included.

## Declaration of competing interest

The authors declare the following financial interests/personal relationships which may be considered as potential competing interests: Muhammad Imran Khan reports financial support was provided by 10.13039/501100004681Higher Education Commission Pakistan. If there are other authors, they declare that they have no known competing financial interests or personal relationships that could have appeared to influence the work reported in this paper.
